# The Promise of Single-Cell RNA Sequencing to Redefine the Understanding of Crohn’s Disease Fibrosis Mechanisms

**DOI:** 10.3390/jcm12123884

**Published:** 2023-06-07

**Authors:** Iona Campbell, Michael Glinka, Fadlo Shaban, Kathryn J. Kirkwood, Francesca Nadalin, David Adams, Irene Papatheodorou, Albert Burger, Richard A. Baldock, Mark J. Arends, Shahida Din

**Affiliations:** 1 Edinburgh Inflammatory Bowel Disease Unit, Western General Hospital, NHS Lothian, Edinburgh EH4 2XU, UK; 2 Edinburgh Pathology, Centre for Comparative Pathology, Cancer Research UK Scotland Centre, Institute of Cancer and Genetics, University of Edinburgh, Crewe Road, Edinburgh EH4 2XU, UK; 3 Edinburgh Colorectal Unit, Western General Hospital, NHS Lothian, Edinburgh EH4 2XU, UK; 4 Department of Pathology, Western General Hospital, NHS Lothian, Edinburgh EH4 2XU, UK; 5 European Molecular Biology Laboratory, European Bioinformatics Institute, EMBL-EBI, Hinxton, Cambridge CB10 1SD, UK; 6 Experimental Cancer Genetics, Wellcome Sanger Institute, Hinxton, Cambridge CB10 1SA, UK; 7 Department of Computer Science, School of Mathematical and Computer Sciences, Heriot-Watt University, Edinburgh EH14 4AS, UK; a.g.burger@hw.ac.uk

**Keywords:** inflammatory bowel disease, Crohn’s disease, fibrosis, single-cell sequencing

## Abstract

Crohn’s disease (CD) is a chronic inflammatory bowel disease with a high prevalence throughout the world. The development of Crohn’s-related fibrosis, which leads to strictures in the gastrointestinal tract, presents a particular challenge and is associated with significant morbidity. There are currently no specific anti-fibrotic therapies available, and so treatment is aimed at managing the stricturing complications of fibrosis once it is established. This often requires invasive and repeated endoscopic or surgical intervention. The advent of single-cell sequencing has led to significant advances in our understanding of CD at a cellular level, and this has presented opportunities to develop new therapeutic agents with the aim of preventing or reversing fibrosis. In this paper, we discuss the current understanding of CD fibrosis pathogenesis, summarise current management strategies, and present the promise of single-cell sequencing as a tool for the development of effective anti-fibrotic therapies.

## 1. Introduction

Inflammatory bowel diseases (IBD) are a group of chronic inflammatory conditions affecting the gastrointestinal tract with a rising health burden globally. In the Western world, a systematic review of population-based studies has shown a stable or decreasing incidence, but the prevalence remains high, surpassing 0.3% in North America, Oceania and much of Europe [1]. In the United Kingdom, a 2018 study showed an overall IBD prevalence of 0.78% (with Crohn’s disease prevalence of 0.28%) and an incidence of 40.8/100,000 patient years in 2017 (13.6/100,000 CD). The incidence per calendar year between 2008 and 2017 was static (annual average percentage change of 14.4%) [2]. In contrast, in newly industrialised countries, the prevalence remains low, but the incidence is increasing [3], mirroring the epidemiological trends seen in the Western world in the 20th century [1]. Therefore, inflammatory bowel diseases will continue to provide significant healthcare challenges throughout the world, and this is set to increase over time. With rising prevalence globally, understanding the disease and developing management strategies have become even more crucial.

Crohn’s disease (CD) is one type of chronic IBD affecting the entire length of the gastrointestinal tract. It is postulated to result from a complex interplay between environmental, immune and intestinal microbiota factors in genetically susceptible individuals. The majority of patients with CD are diagnosed in the second to fourth decade of life, with a further smaller incidence peak in the 50–60 year age group, with no sex-specific distribution [4]. CD follows a relapsing and remitting course and is characterised by chronic transmural inflammation; 35–45% of patients with CD have ileo-colonic disease, 30% have isolated small bowel disease and 20% of patients have colonic disease alone [4]. Around 30% of those with luminal disease are also affected by perianal Crohn’s disease [5]. Extra-intestinal complications (EIM) are also seen commonly, with up to 50% of patients experiencing at least one EIM [6]. The Montreal classification [7] describes the subtypes of CD, taking into account age at diagnosis as well as disease location. It also categorises CD into three broad phenotypes: inflammatory, stricturing and penetrating. The most common presenting phenotype at diagnosis is inflammatory, and it is estimated that 30–50% of those with CD may also have stricturing or penetrating disease at first presentation. CD is a dynamic condition, and over 70% of those with an inflammatory CD phenotype will develop clinically apparent stricturing or penetrating complications within ten years of diagnosis [8,9], with a potential need for surgical intervention. Despite significant advances in therapies targeting inflammatory processes, the need for surgical or endoscopic management of fibrostenotic diseases remains high.

In a study of 1753 CD cases between 2000 and 2017, the cumulative rates of surgery at 1, 5 and 10 years from diagnosis were 6.7% (95% confidence interval [CI], 3.7–10.8%), 16.3% [95% CI, 12.9–20.3%] and 24.4% [95% CI, 20.4–28.7%], respectively [10].

The clinical prediction of which individual with CD will go on to develop this fibrostenotic phenotype is difficult. Furthermore, no specific anti-fibrotic treatment is available for CD. Understanding the pathogenesis of fibrosis is vital to develop new and effective strategies to manage and prevent this significant complication of CD. Single-cell sequencing techniques have provided a new opportunity to map the gastrointestinal tract at a cellular level, thus allowing the identification of specific anti-fibrotic treatment targets.

## 2. Fibrosis Pathogenesis

**Figure 1:**
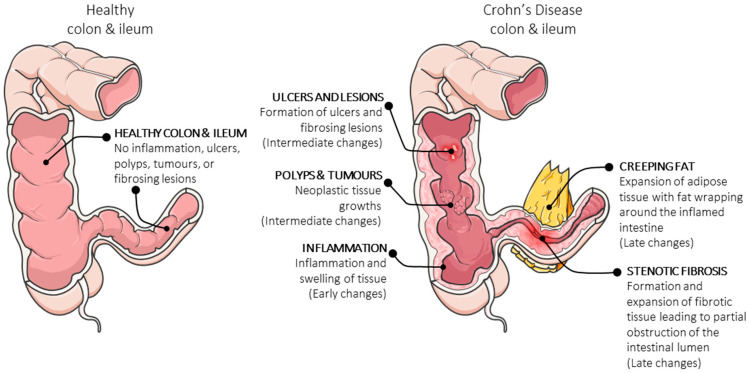
**Illustrative depiction of pathological changes in the colon and ileum during Crohn’s disease.** The diagram shows possible pathological abnormalities that can occur during the early, mid and late stages of Crohn’s Disease. Manifestation of ulcers and inflammatory lesions, formation of polyps and tumours and inflammation progressing to fibrosis of the intestinal wall may progress to fibrostenotic lesions with obstruction of the intestinal lumen.

Crohn’s disease can manifest in a variety of ways. Figure 1 depicts the formation of multiple pathological abnormalities in the colon and ileum. Early inflammation, often with deeply penetrating fissuring ulcers, may progress to fibrostenotic lesions in some patients that necessitate surgical resection [11,12,13]. 

**Figure 2:**
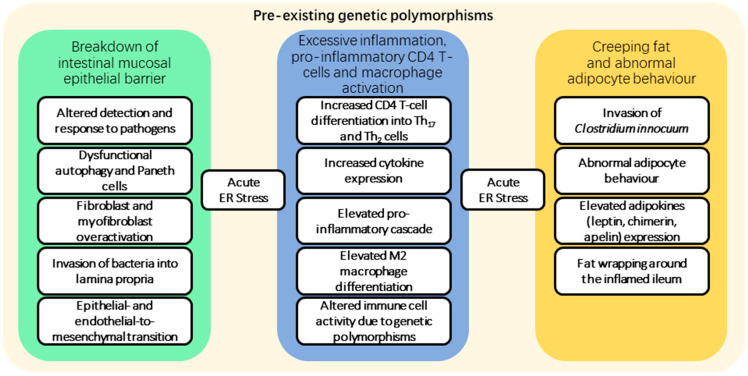
**Overview of the contributory factors leading to fibrostenosis in Crohn’s disease.** Crohn’s disease manifests as a result of interactions between abnormalities in three processes or pathways, usually underpinned by a genetic predisposition in the form of multiple single nucleotide polymorphisms (SNPs) (identified in GWAS studies) that include most commonly: NOD2, ATG16L1, IRGM, LACC1, CARD9, TLRs, LRRK2, MST1, CTLA4, PTPN2 and IL23R, but a long list of others has been recognised. The relevant abnormalities of the three major pathways include (i) intestinal mucosal epithelial barrier breakdown (green), (ii) excessive inflammation with pro-inflammatory CD4 T-cell and macrophage activation (blue), and (iii) formation of creeping fat and abnormal adipocyte behaviour (yellow). These pathway abnormalities interact with each other, resulting in excessive inflammation and fibrosis that, in some patients, may progress to fibrostenosing lesions of Crohn’s disease. The diagram summarises some of the key pathological changes in each of these three pathways that can contribute to the formation of excessive fibrosis. Acute endoplasmic reticulum (ER) stress is a common feature of these three pathways.

Multiple overlapping factors contribute to the formation of fibrostenotic lesions in Crohn’s disease. Figure 2 depicts an overview of the contributory factors involved in the formation of prominent fibrotic tissue in the late stages of Crohn’s disease. In general, a wide range of causative factors of fibrosis have been documented [14,15,16,17,18]. In summary, underlying genetic risk factors (in the form of single nucleotide polymorphisms (SNPs) identified in GWAS studies of Crohn’s disease) together with environmental stimuli interact to contribute to the activation of the inflammatory cascade that leads to the accumulation of fibrosis over time. Immune responses may be triggered within the mucosa and deeper layers of the wall following a breach of the intestinal epithelial barrier, allowing pathogens to enter. This breach is often caused by dysfunctional autophagy and unresponsive Paneth cells, a cause widely known to SNP mutations in NOD2 and ATG16L1, which constitute as the primary cause in around 30% of patients, but this number can vary based on the population [19,20,21]. In the presence of numerous cytokines, particularly interferon gamma, interleukin-12, TGF-beta and interleukin-6, an elevated proinflammatory cascade is activated, which includes CD4 T-cells that can differentiate into pro-inflammatory CD4 Th_2_ and Th_17_ cells that drive inflammatory and fibrogenic processes. The reasons behind these are complex and involve a number of potential SNP mutations in toll-like receptors (TLRs) present on the surface of mononuclear cells, but these interactions are very complex and have been addressed in other studies [22,23,24]. The invasion of specific bacteria and recruitment of inflammatory cells into the mesenteric fat increases cytokine production [25], leading to aberrant adipocyte behaviour and the release of leptin, adiponectin and chemerin, with progression to creeping fat, with adipose tissue expanding and enveloping the inflamed regions of the small intestine, sometimes referred to as fat wrapping [26,27,28,29]. Increasing endoplasmic reticulum stress from the accumulation of unfolded or misfolded proteins inside the affected cells increases signalling pathway activation and cytokine release. The overaccumulation of unfolded proteins leads to the dissociation of immunoglobulin-heavy-chain-binding protein (BiP) that leads to the activation of a number of interlocking pathways, namely, ATF6, IRE1 and PERK pathways [30]. These respond to the presence of unfolded proteins and lead to the degradation of the excess mRNA, inhibit the translation processes, enhance autophagy, and, in extreme cases, lead to cell apoptosis. In Crohn’s disease, these pathways can be deregulated due to the presence of SNPs in critical genes as well as activation of a protein called anterior gradient homologue 2 (AGR2), which can prevent the inhibition of translation and, when released by the cells into the environment, acts as a chemoattractant of monocytes, leading to enhanced inflammation [31,32,33]. 

Macrophages develop into the M2 macrophage phenotype, which adds to the cytokines already released into the intestinal wall [15]. These cytokines activate fibroblasts and myofibroblasts, which generate collagenous fibrotic tissue with an associated extra-cellular matrix (ECM), often together with the in-growth of granulation tissue capillaries as part of a wound healing response [34,35]. Some epithelial cells around the site of breach of the epithelial barrier may transform into mesenchymal-like cells in a process of epithelial-to-mesenchymal transition [36,37,38,39]. Furthermore, some endothelial cells from either the granulation tissue capillaries or pre-existing vasculature may also undergo a similar process of endothelial-to-mesenchymal transition [40,41]. Variable combinations of these changes result in fibroblast activation with ECM release, and increasing fibrotic tissue formation contributes to fibrostenosing lesion formation in Crohn’s disease.

## 3. Current Treatment Strategies

Currently, no specific anti-fibrotic therapy exists for CD, so treatment is currently aimed at managing the complications of stricturing disease. The CONSTRICT (Crohn’S disease anti-fibrotic stricture therapies) expert consensus group [9] has published suggestions for diagnostic criteria for small bowel CD-related strictures. Magnetic resonance (MR) and computer tomography (CT) enterography are suggested as the preferred diagnostic tools for stricture assessment. Overall, MR enterography is favoured due to its lack of radiation exposure. Small bowel ultrasound (US) is another emerging imaging technique that appears to be able to accurately identify small bowel CD-related strictures [42]. Endoscopic evaluation requires adequate bowel preparation, which can be challenging to achieve in patients with CD, with evidence that those with active CD experience more abdominal pain during bowel preparation, which is a predictor of poor preparation [43]. When good bowel cleansing is achieved, strictures may be visualised endoscopically, but assessment is limited by its superficial mucosal views, lack of transmural assessment and inability to traverse the strictured bowel [13].

While techniques to identify the presence of a stricture are well established, assessing the composition (inflammatory versus fibrotic) is more challenging, and meta-analysis in this area is lacking [13]. However, accuracy may improve when techniques that assess small bowel motility are routinely integrated into clinical practice [44,45]. In surgical resections, most stricture specimens have both inflammatory and fibrotic components [9]. 

If there is clinical suspicion of an inflammatory component of the stricture, anti-inflammatory therapy, typically with steroids acutely followed by anti-tumour necrosis factor (anti-TNF) medication, is indicated. A recent prospective and observational trial, the CREOLE study, evaluated the efficacy of adalimumab in 97 patients with symptomatic small bowel strictures. In this group, 64% achieved success at 24 weeks (did not require other anti-inflammatory drugs or endoscopic/surgical intervention), and at follow-up (median time 3.8 years), 45.7 ± 6.6% of those who had achieved success at 24 weeks remained in prolonged success at four years [46]. There is growing interest within the clinical community in the ‘window of opportunity’ in CD [47], which describes the concept of commencing aggressive treatment early in the disease course before bowel damage occurs, as well as the concept of treatment to target (such as mucosal healing) and tight monitoring of both drug levels and biochemical markers of inflammation, such as faecal calprotectin.

In addition to maximising the efficacy of currently established treatments, new medical therapies are being investigated, including mesenchymal stem cells [48] and small molecules with antifibrotic properties [49], although it will be some time before they reach clinical practice.

In contrast, for strictures suspected to be primarily fibrotic in nature, surgery or endoscopic intervention remains the mainstay of treatment. The current approaches are summarised in Table 1. Endoscopic intervention generally involves either balloon dilatation, endoscopic stricturotomy or stenting. It is generally suitable to consider endoscopic therapy for short (<5 cm), straight strictures without penetrating complications or deep ulceration [50,51]. Endoscopic balloon dilatation (EBD) is the most commonly used technique, with technical success rates (defined as the ability of the endoscope to pass through the stricture following dilatation) of over 80% [52] and high rates of short-term symptomatic improvement [51,52]. However, the risk of complications, including luminal perforation, is around 3%, and this risk increases with increasing stricture length [50,52]. Symptomatic recurrence is also high, and there is likely to be a requirement for further dilatation or surgery [50].

Endoscopic stricturotomy is a newer technique that is not yet widely practised, and is associated with a lower perforation rate and lower stricture recurrence rates compared to balloon dilatation, but with a higher bleeding risk [53]. Endoscopic stenting with a self-expanding metal stent (SEMS) is another potential strategy for the management of CD-related strictures, but further studies are needed to assess the effectiveness of this against more well-established techniques. A randomised trial of 80 patients in 19 Spanish centres demonstrated a similar safety profile for SEMS versus EBD, but EBD was found to be more effective overall [54]. Surgery can, in some circumstances, be an appropriate first-line management option for the management of CD-related strictures. Indications for a surgical approach over endoscopic management include fistulating disease, abscess formation or significant pre-stenotic dilatation proximal to the stricture. First-line management should also be considered if there is any concern regarding malignancy.

The main surgical options include resection and stricturoplasty. The choice of which technique to use depends on, amongst others, the site, length and number of strictures. The European Crohn’s and Colitis Organisation (ECCO) and the European Society of Proctology (ESCP) published consensus guidelines on surgery for CD in 2018. The guidelines recommend that, where technically feasible, stricturoplasty should be considered first-line surgical management, particularly when long segments of the small bowel are affected (to minimise the risk of short-bowel syndrome), in recurrent strictures at ileocolic anastomotic sites, and where there is no complicating factor such as an associated abscess [55].

In localised stricturing ileo-caecal Crohn’s disease, first-line surgical management is with either a laparoscopic ileo-caecal resection or stricturoplasty, unless there are perforating complications, in which case resection is required [55]. 

There remains a high risk of recurrence following surgical intervention, and thus the risk of further surgeries also remains; with repeated resections, eventually, there is a risk of short-bowel syndrome, with major implications for nutrition and quality of life.

In the BACARDI study [56], a risk model was suggested to identify those most at risk of requiring surgery and those who may benefit from ongoing medical management or endoscopic intervention for stricturing CD.

However, none of these options provide definitive, recurrence-free treatment for Crohn’s fibrosis; thus, the need for therapies specifically targeting the pathways leading to fibrosis is urgently required. A recent paper by Lin et al. [57] highlights the developed anti-fibrotic therapies that already exist for conditions affecting the lung, kidney, skin and liver, and identifies several potential therapies that may have a transferrable role in managing Crohn’s fibrosis. These include targets such as growth factor modulators, inflammation modulators, intracellular enzymes and kinases, extracellular matrix (ECM) modulators, renin-angiotensin system (RAS) modulators and 5-hydroxy-3-methylglutaryl-coenzyme A (HMG-CoA) reductase inhibitors. While these may present promising opportunities for further study, a greater understanding of the gut at a cellular level, particularly in relation to fibrosis, is essential to develop novel therapeutic options for CD specifically.

**Table 1:** Surgical and endoscopic approaches for stricture management of CD.

Intervention	Indications	Technical Success Rates	Complications	Recurrence
**Endoscopic balloon dilatation**	- Short, straight strictures <5 cm - No penetrating complications or deep ulceration - No evidence of malignancy	- >80% (the ability of the endoscope to pass stricture following dilatation) [52]	- 3% perforation risk (increases by 8% for every 1 cm increase in stricture length) [52]	- 52% require repeat dilatation and 30% require surgical intervention at 12 months [50] - 73% require repeat dilatation and 42% require surgery at 24 months [52]
**Endoscopic stricturotomy**	- Not yet widely practiced	- >90% immediate technical success rate in retrospective studies [58,59,60]	- Lower perforation rate but higher bleeding rate than balloon dilatation [53]	- Small retrospective studies report a 9–15% subsequent need for surgical intervention [59,60]
**Endoscopic self-expanding metal stent**	- Not yet widely practiced	- >90% technical success rate in small retrospective studies - with 60–80% initial symptomatic improvement [61,62]	- Safety profile similar to EBD in one randomised trial; further studies needed [54]	- 49% of patients required further intervention at 12 months in one randomised trial; further studies needed [54]
**Surgical stricturoplasty**	- Long strictures to minimise the risk of short bowel - Ileo-colonic anastomotic strictures	NA	- Peri-operative complication rate averages 13% [63]	- Low site-specific recurrence rate (2–5% at 10 years) [55]
**Surgical resection**	- Complicated disease with perforation or abscess, or concern regarding malignancy - Localised ileo-caecal disease	NA	- Dependent on multiple factors including the extent of surgery and approach required - Risk of short bowel with multiple/extensive resections	- 25% recurrence rate in a meta-analysis of six studies [64]

## 4. Single-Cell Sequencing

Conventional research approaches investigating Crohn’s disease rely on the superficial assessment of endoscopic mucosal biopsies or on the large amount of data generated by surgical resection specimens. However, their ability to decipher the complex cellular networks and environmental topography, which may help identify new therapeutic targets, is limited. Single-cell techniques have provided new tools for investigating cellular changes in tissues, including heterogeneity of cellular composition, the differential abundance of cell (sub-)populations, along with alterations in cell states between normal and diseased conditions. A number of single-cell sequencing technologies have been designed for the investigation of the genome, transcriptome, epigenome (chromatin accessibility, DNA methylation, histone modifications and chromosome structure) and proteome (surface marker expression). In addition, spatial transcriptomics allows the correlation of such data with the two-dimensional location of cells within tissue sections [65,66,67,68]. 

Recent studies related to Crohn’s disease have focused on single-cell transcriptomics (scRNA-seq), gut microbiome and dysbiosis [69]. It has been established that Crohn’s disease usually involves pre-existing genetic polymorphisms that interact with environmental triggers. A number of studies have explored the most common microbiota composition present in Crohn’s disease patients and found organisms such as *Escherichia*, *Shigella* or *Atlantibacter* species [70]; however, this often correlates with the patient’s original geographical disease location [71]. Certain bacterial strains such as *Clostridium innocuum* have been shown to be involved in deep tissue penetration, invading the mesenteric fat surrounding ileal tissue, which has been proposed to lead to pro-inflammatory and pro-adipogenic responses, contributing to the formation of “creeping fat” or “fat-wrapping” [25]. 

The role of the immune system in Crohn’s disease biology, specifically that of pro-inflammatory CD4 T-cells, has been studied by scRNA-seq, with a recent focus on CD8 T-cells and populations of natural killer T (NKT) type II cells identified in Crohn’s disease [72]. The presence of CD8 T-cells that express surface markers CD39^+^ and PD-1^+^ was associated with disease progression, with the exhaustion of CD39^+^ PD-1^+^ CD8 T-cells correlating with remission [73,74], although the mechanisms for this require further investigation [75]. The use of scRNA-seq for studying mononuclear phagocyte populations in the lamina propria of the intestines has shown changes in macrophage and dendritic cell subtypes during inflammatory bowel disease inflammation [76]. 

Furthermore, a recent study by Mukherjee et al. [77] examining scRNA-seq in stricturing disease showed increased fibroblast heterogeneity, particularly in the mucosa and submucosa, in areas of stricture compared to non-strictured bowel. This suggests that upregulation of fibroblast-specific markers in areas of stricturing—for example, Cadherin-11, a profibrotic cell surface receptor expressed in these fibroblasts—may play a major role in the development of fibrosis.

Single-cell sequencing methods have led to an increased understanding of the heterogeneous cellular changes in Crohn’s disease, which may shed new light on potentially novel therapeutic targets and mechanisms of resistance to existing therapies [78]. The Gut Cell Atlas (www.gutcellatlas.org, accessed on 7 May 2023) is a valuable resource for profiling the gut cellular heterogeneity in healthy individuals, with applications to the study of Crohn’s disease in comparison with a control [79,80]. The integration of single-cell samples is possible due to machine-learning techniques [81] and allows population-specific differential gene expression profiling between conditions [82,83], the discovery of new cell types and the capture of differentially abundant states [84]. Evolutionary trajectories can be inferred using computational methods for pseudo-time ordering [85]; when samples at multiple disease stages are available, this information can be integrated into the model [86] to improve the predictions. 

## 5. Spatial Analysis

For fibrosing CD, the ability to spatially characterise the dynamic pathogenesis at a cellular level will be critical in facilitating targeted drug design. Single-cell sequencing technologies have the power to redefine disease mechanisms as seen in hepatic fibrosis; therefore, unbiased gene expression analysis may identify rare cell types, transforming our understanding of CD-related fibrosis. Specifically, novel highly multiplexed microscopy techniques that establish gene expression at cellular resolution across histological sections can reveal the spatial organisation of detailed cellular activity within tissues, thus enabling these cell types to be located within their functional groups. The collection of spatial data is crucial to precisely pinpoint the differences in the severity of the disease progression, depending on the location of its expression. This type of detailed spatial analysis is possible when the gut location is precisely recorded during tissue specimen collection.

**Figure 3:**
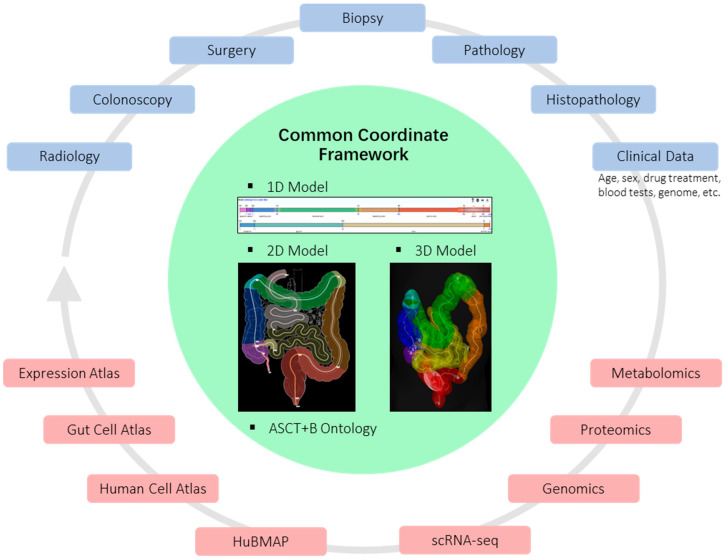
**Clinical and research data integration.** The Common Coordinate Framework depicted within the centre is a series of spatially interoperable models in 1D, 2D and 3D [89], combined with anatomy and cell-type ontologies. Spatially annotated data using any of the CCF representations can be visualised within any other representation, cross-queried and compared. Each sample/observation or data type can be annotated using the CCF models, and thus they become integrated with all other spatially mapped data. This integration is depicted by a continuous grey arrow linking the boxes. The upper, blue-bounded boxes show different clinical and patient data that could be mapped; the lower, red-bounded boxes are large data sources and atlas research programmes that deliver relevant data and analysis tools. For open access, all clinical data are fully anonymised, and the clinical data could include all that is relevant to interpreting the observations and comparisons with other cases. The “Clinical Data” input box includes some possible inputs but could extend to a more detailed clinical history, if relevant.

Inherent anatomical variations, the effects of chronic disease and previous surgery make it challenging to accurately determine the origin of tissue samples, limiting data interpretation and clinical translation [87]. This has been recognised within the global Human Cell Atlas (HCA) initiative [88] and in the gut context with the development of the Human Gut Cell Atlas (HGCA) promoted by NIH-funded HuBMAP and Helmsley Trust HGCA programmes. A roadmap for the human developmental cell atlas [88] and a proposal for a focused gut-specific Common Coordinate Framework (CCF) have been published [89] and illustrated in Figure 3. In brief, a common coordinate framework is being established to provide a mechanism for data integration and analysis that will allow appropriate cross-study analysis and comparison. If standard protocols for mapping data of all types can be adopted, the data can be compared, analysed and visualised for spatial dependencies hitherto not understood or discovered. In the context of the CCF shown in Figure 3, mapping can be performed for any of the model representations (1D–3D), visualised within any of the models; therefore, the data can become accessible for machine learning or AI approaches for the discovery of spatial patterns.

## 6. Collaborative Approaches

Zilbauer et al. [90] have published their vision to achieve these goals and develop a common coordinate framework. They outline the requirements for a structured and coordinated approach across the scientific community to allow successful collaborative efforts to develop this invaluable tool.

They highlight several areas for development. One such area is that of ensuring tissue sampling of the gut for accurate mapping including all anatomical areas, suggesting the use of resection and deceased tissue specimens, as mucosal biopsies—although easily obtained during routine clinical practice—capture only superficial layers of the gut mucosa, and are generally confined to the large bowel and terminal ileum, or upper GI tract.

Furthermore, they recommend a template for metadata collection that should be used by the scientific community to standardise data collection across studies, allowing for the comparability and integration of datasets. They also review the existing data portals available for interrogating and combining datasets and suggest this as a further area for development.

Several of these challenges are currently being addressed by our group, including standardising single-cell isolation from fibrotic intestinal tissue and developing platforms to integrate clinical, histological, radiological and computational data. 

A challenge to the clinical community is to adopt and introduce standard reporting practices, which are more consistent and rigorous methods of anatomical and tissue annotation to precisely localise and reveal the spatial aspects of cellular and disease heterogeneity. 

## 7. Conclusions

At present, no effective anti-fibrotic treatment exists to manage and prevent Crohn’s disease fibrosis. In fact, the term “Crohn’s Disease” has been debated over the last two decades [68] due to the wide spectrum of genetic causes and environmental triggers that result in varying disease progression in patients, and thus should be termed as “Crohn’s Diseases” to reflect this [91]. As the molecular characterisation and understanding of Crohn’s disease evolve, the Montreal Classification may need to be reviewed and expanded to encompass the novel findings. To achieve this, a cross-disciplinary approach between clinicians, surgeons, radiologists, pathologists and data and computational scientists will be critical in fully understanding Crohn’s disease, the formation of fibrosis and the development of more effective treatment strategies. 

The Common Coordinate Framework can be used to address the necessity of developing new treatments. The vast amount of publicly available multi-omics data, if mapped with available atlas data and clinical data, could benefit clinicians and patients by creating more efficient ways of visualising and analysing the available data, and thus help identify novel biomarkers for the early detection of fibrosis and personalised treatments, and thus avoiding drug exposure if the phenotype is associated with an increased likelihood of surgery, preventing collateral tissue damage and reducing the possibility of an elevation of the symptom severity.

## Data Availability

Not applicable.
